# Estimates of the Dietary Glycemic Index and Load in a Representative Sample of the Greek Population

**DOI:** 10.3390/nu17223596

**Published:** 2025-11-18

**Authors:** Georgia Vourli, Livia Augustin, Carlo La Vecchia, Eleni Peppa, Antonia Trichopoulou

**Affiliations:** 1 Center for Public Health Research and Education, Academy of Athens, Alexandroupoleos 23, 11527 Athens, Greece; epeppa@academyhealth.gr (E.P.); atricho@academyhealth.gr (A.T.); 2 National Cancer Institute, Istituto Nazionale Tumori, IRCCS “Fondazione G. Pascale”, 80131 Naples, Italy; l.augustin@istitutotumori.na.it; 3 Department of Clinical Sciences and Community Health, Università degli Studi di Milano (“La Statale”), 20137 Milan, Italy; carlo.lavecchia@unimi.it

**Keywords:** glycemic index, glycemic load, Mediterranean diet, demographic, age, sex, BMI, smoking, education, SES

## Abstract

Introduction: The dietary glycemic index (GI) and glycemic load (GL) are dietary indicators of how carbohydrate-containing foods affect blood glucose. While the Mediterranean diet’s glycemic impact has been explored, data specific to the Greek population remain limited. This study aims to assess the dietary GI and GL in the HYDRIA survey of a representative sample of Greek adults. Methods: HYDRIA was conducted from June 2013 to December 2014 and collected nationally representative data, including detailed dietary information. GI and GL were analyzed by age, sex, BMI, education, employment, smoking status, and Mediterranean diet adherence. Multivariate analysis was used to identify independent predictors of GI and GL. Results: The analysis included 3951 adults (52% females; median age: 49 years). The average GI was 59.7 (95% CI: 59.4–60.0) and the mean GL 101.7 (95% CI: 99.4–104.0). Males had significantly (*p* ≤ 0.001) higher mean GI and GL (61.2, 95% CI: 57.4–64.7 and 112.9, 95% CI: 82.1–151.1, respectively) than females (59.2, 95% CI: 55.6–62.8 and 81.9, 95% CI: 62.2–107.8, respectively). Older age (≥55 years) and higher education were associated with lower GI and GL in both sexes. Current smoking was associated with higher GI and GL, particularly in males. Among males, medium and high adherence to the Mediterranean diet was associated with lower GI and GL. This relationship was not observed in females. Discussion: These findings emphasize the role of demographics and lifestyle factors in determining differences in dietary GI and GL of the Greek population. The Mediterranean diet appeared to mitigate the dietary GI and GL mainly in males, suggesting effects that merit further investigation.

## 1. Introduction

The quantity and quality of dietary carbohydrates are the main dietary factors that affect glycemic responses. The glycemic index (GI) and glycemic load (GL) are established characteristics of the food, and not of the subjects, used to assess the impact of carbohydrate containing foods and meals on blood glucose levels [1,2]. The GI concept is defined as the area under the blood glucose response curve following the consumption of a fixed amount of 50 g of available carbohydrate from a test food, expressed relative to the response elicited by a reference food, typically a glucose solution or white bread [3,4,5]. The higher the dietary GI or GL the higher the glycemic and insulinemic responses [3,6,7]. In 1997, Salmeron proposed the GL which accounts for both the quality (GI) and quantity of carbohydrates in a serving of food [8]. Therefore, while GI reflects how rapidly a carbohydrate turns into glucose independently of portion size, GL reflects the total glycemic impact of a standard portion of that food [9]. Both GI and GL can be used to inform dietary decisions related to carbohydrate needs and to prevent harmful health outcomes. Dietary GI and GL are characteristics of the food, have direct effects on carbohydrate metabolism and on long-term effects on blood glucose control, insulin sensitivity and ultimately on the risk of diabetes, obesity and cardiovascular disease [10,11,12].

According to the Food and Agriculture Organization of the United Nations(FAO) and the World Health Organization (WHO), estimating GI and GL is essential for assessing carbohydrate quality and guiding decisions to optimize metabolic health [4]. Therefore, it is important to have relevant data and report population-level GI and GL values. The GI of a particular food or meal is influenced by the type of carbohydrate present (starch vs. mono-or di-saccharides), food processing, cooling after cooking, liquid or dry form, but also by other dietary components, such as type and amount of dietary fiber, the presence of lipids, short chain fatty acids and proteins, that regulate carbohydrate digestion and absorption rates as well as insulin secretion [13,14,15,16]. The dietary GI, however, remains a property of the food and not of the subject consuming it.

Dietary patterns vary considerably across populations in carbohydrate quantity and the combinations of foods typically consumed. For instance, the Mediterranean diet is characterized by carbohydrate intake accompanied by high fiber and unsaturated fat content, whereas traditional dietary patterns in East Asia or Latin America include distinct carbohydrate sources and food combinations [17,18,19,20]. Consequently, understanding the specific dietary patterns of a population is essential for accurately interpreting GI and GL values and for tailoring nutrition recommendations that take into account specific dietary habits and preferences.

People living in the Mediterranean region share broadly similar dietary patterns, collectively known as the Mediterranean diet [21]. However, regional variations in food choices and preparation methods influence the overall GI and GL of the diet. In Greece, for example, the traditional diet is characterized by high consumption of olive oil, cereals, legumes, vegetables and fruit, and therefore foods may contain a high proportion of fat and dietary fiber. These features would lower the overall impact of high GI foods on postprandial glycemia and insulinemia and benefit satiety, and long-term glycemic control and body weight [5,22,23]. In addition, in the traditional Mediterranean diet, fruits are often consumed at the end of a meal, which does not produce the same glycemic spike as when fruit is eaten on its own, even in the case of high GI fruits such as grapes, watermelon, or figs. Fructose in fruit appears to have a catalytic effect, potentially enhancing the overall metabolism of carbohydrates within the meal [24].

Similarly, in Spain, the typical dietary pattern closely resembles the Greek diet, but with higher quantities of rice and nuts [25,26]. In contrast, while the Italian diet shares core Mediterranean elements, it tends to include more pasta made of durum wheat semolina which has a low GI [27,28].

Although the glycemic impact of the Mediterranean diet has been studied, most notably through large-scale trials like Prevention with Mediterranean Diet (PREDIMED) [25], there remains a notable gap in the literature regarding the dietary GI and GL of Mediterranean countries, specifically for the Greek population. Given the established associations of GI and GL with a wide range of health outcomes, our study aims to provide a detailed assessment of these measures in a representative sample of adult permanent residents of Greece.

## 2. Methods

The nationally representative survey HYDRIA was performed in Greece from June 2013 to December 2014 and included 4011 adult participants coming from 51 regional areas (prefectures) of the 13 regions of the country. It was based on the general census on population and housing of 2011. It was designed as two-staged stratified random sampling, with the primary sampling unit being the municipal/local community (1st stage) and the final unit being the individual (2nd stage). This allows to produce results generalizable to the adult Greek population. Data were collected in accordance with the guidelines of the Helsinki Declaration and the national data protection legislation. Data collection was performed following the recommendations of the European Food Safety Authority (EFSA) [29] and the European Health Examination Survey (EHES) [30], allowing for intercountry comparisons. Details regarding HYDRIA’s study tools and methods have been published [31]. All procedures involving human subjects/patients were approved by the Hellenic Data Protection Authority in 2012, in order to establish and operate a registry of sensitive personal data files under Law No. 2472/1997. All participants provided signed informed consent prior to enrollment.

Dietary intake was assessed using two non-consecutive 24-h recalls (24 HR) per participant, and a non-quantitative food propensity questionnaire (FPQ). In the current analysis, only the two 24 HR were considered.

To derive GI values for each food item, a literature review was conducted. The following sources were included: University of Sydney GI database (http://www.glycemicindex.com/foodSearch.php), Diogenes GI Database, British values, USDA database Glycemic Index and Glycemic Load of 3775 Foods (https://www.foodhealth.info/, accessed on 1 March 2017), Harvard database (https://www.health.harvard.edu/diseases-and-conditions/glycemic-index-and-glycemic-load-for-100-foods, accessed on 1 March 2017), Montignac Method (http://www.montignac.com/en/search-for-a-specific-glycemic-index/#tab_low, accessed on 1 March 2017), and finally Sugar and sweetener guide (http://www.sugar-and-sweetener-guide.com/sweetener-values.html, accessed on 1 March 2017). Food items containing negligible amounts of carbohydrates or that do not increase blood glucose levels (mainly meat and fish, fats, and eggs) were not assigned any GI values.

The overall dietary glycemic Index per day was calculated as ∑inGIi×CHOi∑inCHOi and daily glycemic load as $\sum_{i}^{n}{GI}_{i}\times {CHO}_{i}$, for the food items 1 to *n* (*n* is the number of foods eaten per day), where *GI*_i_ is the reference glycemic index for each food item and the carbohydrate content in food i (grams per day). The *GI* for each participant was estimated as the average of the two GIs from the two 24 HR.

Participants who did not complete at least one 24 HR food questionnaire were not included in the analysis.

### Data Analysis

To account for the HYDRIA’s design, sampling and post-stratification weights were applied throughout the statistical analysis. Categorical characteristics are summarized as absolute and weighted relative frequencies (*n* and %), while comparisons between groups were performed using weighted chi2 tests. Subject characteristics included age group, sex, BMI category, educational level, employment status, smoking status and adherence to the Mediterranean Diet. The adherence to the traditional Mediterranean diet (Mediterranean diet score) was assessed using a score developed by Trichopoulou [17]. A value of 0 or 1 was assigned to each of the nine indicated components with the use of the sex-specific median as the cutoff. For beneficial components, such as vegetables, legumes, fruits, nuts, cereal, and fish, when consumption was below the median, it was assigned a value of 0, while those whose consumption was at or above the median were assigned a value of 1. For detrimental components like meat, poultry, and dairy products, intakes below the median were assigned a value of 1, while those above the median were assigned a value of 0. For ethanol, a value of 1 was assigned to men who consumed between 10 and 50 g per day and to women who consumed between 5 and 25 g per day. Finally, for fat intake, we used the ratio of monounsaturated to saturated fatty acids (MUFA and SFA, respectively) rather than the ratio of polyunsaturated to saturated fatty acids (PUFA) because in Greece MUFA are used in much higher quantities than PUFA. Thus, the total Mediterranean diet score ranged from 0 (minimal adherence to the traditional Mediterranean diet) to 9 (maximal adherence) and the score was then categorized in three groups: 0–3, 4–5, 6–9 for low, intermediate and high adherence, respectively [17]. GI and GL were summarized as means and corresponding 95% confidence intervals (95% CI), overall and by participants’ characteristics. Comparisons of their values between groups were performed using weighted *t*-tests. To identify independent factors affecting GI and GL, weighted multivariate regression models were fitted. Analyses were performed separately for males and females, to account for different dietary habits by sex. All multivariable models were adjusted for total energy intake, as well as the amount of carbohydrates and fat consumed. Statistical analyses were performed with Stata 11.0.

## 3. Results

HYDRIA included 4011 participants of whom 3951 had complete dietary data and were included in this analysis. The sample was balanced regarding sex (52% females and 48% males), and the median participants’ age was 49 years (IQR 35.4–65 years) in the overall sample and similarly across sexes. Overweight (37.4%) and obesity (34.8%) were the most common BMI categories, with obesity being more prevalent among females and overweight more common among males. Underweight status was rare, especially among males. Educational level and employment status differed by sex: 44.0% of females had low education versus 34.9% of males, while 68.3% of females were not employed compared to 48.0% of males. Smoking patterns also varied; more females were never smokers, while males had higher rates of current and former smoking. Adherence to the Mediterranean diet was low overall (44.9%), with poorer adherence in females (Table 1).

The mean GI was 59.7 (95% CI: 59.4 to 60.0) and the mean GL 101.7 (95% CI: 99.4 to 104.0). Individuals in higher tertiles of both GI and GL tended to consume higher quantities of energy and nutrients and lower quantities of dietary fiber (Table 2).

**Table 2:** Nutrient profile by tertiles of Glycemic Index (A) and Glycemic Load (B).

(A)	Tertile 1	Tertile 2	Tertile 3
	(Mean GI: 53)	(Mean GI: 60)	(Mean GI: 66)
*Energy* (kcal)	1658.1 (1606.5–1709.7)	1890.3 (1840.1–1940.5)	1996.8 (1933.3–2060.2)
*Protein* (g)	63.4 (61.4–65.5)	70.5 (68.3–72.8)	76.3 (73.4–79.2)
*Carbohydrates* (g)	147.6 (142.5–152.7)	173.8 (168.6–179)	183.6 (177.1–190.1)
*Fat* (g)	80.4 (77–83.8)	88.9 (86.2–91.5)	92.3 (89–95.7)
*SFA* (g)	25.2 (24.2–26.3)	27.7 (26.8–28.5)	28.7 (27.5–29.8)
*MUFA* (g)	39.1 (37.2–41.1)	42.6 (41.2–44.1)	43.7 (42–45.4)
*PUFA* (g)	10.2 (9.7–10.8)	12.3 (11.8–12.8)	13.4 (12.7–14.1)
*Cholesterol* (mg)	184.6 (174.6–194.5)	213.4 (200.7–226.1)	225.4 (212.6–238.2)
*Fiber* (g)	17.3 (16.6–18)	17.3 (16.7–18)	15.8 (15.2–16.3)
**(B)**	**Tertile 1**	**Tertile 2**	**Tertile 3**
	**(mean GL: 55)**	**(mean GL: 92)**	**(mean GL: 158)**
*Energy* (kcal)	1244.5 (1198.8–1290.2)	1746 (1706.6–1785.4)	2554.5 (2500.5–2608.4)
*Protein* (g)	51.5 (49.4–53.6)	66 (63.7–68.2)	92.8 (90.5–95)
*Carbohydrates* (g)	95.7 (93.8–97.7)	155 (153.5–156.4)	254.2 (248.6–259.8)
*Fat* (g)	63.6 (60.5–66.7)	84.1 (81.2–87)	113.9 (110.7–117.2)
*SFA* (g)	19.4 (18.5–20.3)	26.1 (25.2–27)	36 (35–37.1)
*MUFA* (g)	31.5 (29.8–33.2)	40.6 (39–42.3)	53.4 (51.7–55.1)
*PUFA* (g)	8.2 (7.6–8.7)	11.4 (10.8–12)	16.3 (15.7–16.9)
*Cholesterol* (mg)	165.8 (154.6–176.9)	196.4 (185.8–207)	261.2 (248.7–273.7)
*Fiber* (g)	11.9 (11.4–12.3)	16.1 (15.6–16.5)	22.5 (21.7–23.3)

In univariate analysis, both GI and GL were significantly higher in males than in females (61.2 vs. 59.2 and 112.9 vs. 151.1 respectively; *p* = 0.001 for both indices), while they decreased with age in both sexes (*p* < 0.001) (Table 3 and Table 4).

**Table 3:** Sex-Stratified Glycemic Index Values Across Subgroups.

	Females (*n* = 2115)			Males (*n* = 1836)		
	N	Glycemic Index (Mean (95% CI))	*p*-Value	N	Glycemic Index (Mean (95% CI))	*p*-Value
**Overall**		59.2 (55.6–62.8)			61.2 (57.4–64.7)	
*Age (years)*			0.001			<0.001
18–44	935	59.6 (59.2 to 60.1)		798	61.2 (60.7 to 61.7)	
45–64	763	58.5 (57.9 to 59.0)		648	60.8 (60.1 to 61.4)	
65+	417	58.4 (57.4 to 59.4)		390	58.9 (58.2 to 59.6)	
*BMI*			0.046			0.249
Underweight (<120 kg/m^2^)	105	60.6 (59.3 to 61.9)		16	61.8 (59.3 to 64.4)	
Normal (20–24.9 kg/m^2^)	646	59.1 (58.5 to 59.6)		361	61.2 (60.4 to 61.9)	
Overweight (25–29.9 kg/m^2^)	666	59.0 (58.3 to 59.7)		825	60.4 (59.9 to 61.0)	
Obese (≥30 kg/m^2^)	670	58.6 (57.9 to 59.3)		596	60.2 (59.6 to 60.9)	
*Educational level*			0.038			<0.001
Low	644	59.0 (58.3 to 59.8)		472	60.8 (60.1 to 61.4)	
Intermediate	877	59.2 (58.7 to 59.6)		736	61.0 (60.5 to 61.5)	
High	594	58.2 (57.7 to 58.8)		628	59.4 (59.0 to 59.9)	
*Employment Status*			0.022			0.004
Employed	1408	58.7 (58.2 to 59.2)		902	60.0 (59.6 to 60.5)	
All other	707	59.5 (58.9 to 60.0)		934	61.0 (60.5 to 61.5)	
*Smoking*			0.007			<0.001
Never	1080	58.8 (58.2 to 59.3)		567	60.0 (59.4 to 60.6)	
Ex smoker	290	58.1 (57.2 to 59.0)		550	59.4 (58.8 to 60.1)	
Current smoker	741	59.5 (59.0 to 60.0)		711	61.7 (61.2 to 62.3)	
*Med. Diet Adherence Category*			0.449			0.002
Low	997	59.2 (58.7 to 59.6)		728	61.4 (60.8 to 62.0)	
Med	856	58.8 (58.1 to 59.5)		766	60.0 (59.5 to 60.6)	
High	262	58.5 (57.2 to 59.8)		342	59.7 (58.9 to 60.6)	

**Table 4:** Sex-Stratified Glycemic Load Values Across Subgroups.

	Females (*n* = 2115)			Males (*n* = 1836)		
	*n*	Glycemic Load (Mean (95% CI))	*p*-Value	*n*	Glycemic Load (Mean (95% CI))	*p*-Value
		81.9 (62.2–107.8)			112.9 (82.1–151.1)	
*Age (years)*			<0.001			<0.001
18–44	935	96.2 (93.0 to 99.4)		798	135.3 (129.0 to 141.5)	
45–64	763	85.6 (82.5 to 88.8)		648	114.2 (107.6 to 120.7)	
65+	417	73.4 (68.4 to 78.4)		390	90.0 (84.9 to 95.1)	
*BMI*			<0.001			<0.001
Underweight (<20 kg/m^2^)	105	114.9 (98.4 to 131.4)		16	161.0 (139.9 to 182.1)	
Normal (18.5–24.9 kg/m^2^)	646	94.1 (90.2 to 98.1)		361	134.6 (129.0 to 140.3)	
Overweight (25–29.9 kg/m^2^)	666	83.6 (80.1 to 87.1)		825	119.8 (113.5 to 126.1)	
Obese (≥30 kg/m^2^)	670	80.5 (76.3 to 84.7)		596	106.6 (101.0 to 112.3)	
*Educational level*			<0.001			<0.001
Low	644	79.1 (74.5 to 83.7)		472	107.1 (101.0 to 113.3)	
Intermediate	877	91.4 (88.3 to 94.5)		736	124.8 (118.6 to 131.0)	
High	594	94.7 (90.6 to 98.8)		628	122.2 (116.4 to 127.9)	
*Employment Status*			<0.001			<0.001
Employed	1408	82.3 (79.6 to 85.0)		902	110.5 (104.9 to 116.0)	
All other	707	96.1 (92.1 to 100.1)		934	125.0 (120.0 to 130.0)	
*Smoking*			0.429			0.005
Never	1080	85.5 (82.5 to 88.5)		567	118.9 (113.2 to 124.6)	
Ex smoker	290	87.1 (80.6 to 93.6)		550	108.1 (100.9 to 115.3)	
Current smoker	741	88.3 (84.5 to 92.2)		711	125.2 (118.7 to 131.6)	
*Med. Diet Adherence Category*			0.001			0.409
Low	997	83.8 (80.8 to 86.8)		728	114.9 (108.5 to 121.3)	
Med	856	86.7 (83.0 to 90.3)		766	120.5 (114.7 to 126.4)	
High	262	97.4 (90.6 to 104.3)		342	119.3 (113.0 to 125.7)	

Both GI and GL decreased with age and being employed, in both sexes. Smoking status was significantly associated with GI and GL; current smokers had the highest GI values and former smokers the lowest, overall and in both sexes separately, while for GL, this pattern was significant only in males, where current smokers had higher values than former or never smokers. Adherence to the Mediterranean diet was inversely associated with GI in males but not in females, while the opposite pattern was observed for GL, which was higher in females with greater adherence and showed no significant association in males.

The effect of sex on both GI and GL remained significant in the presence of all potential confounders.

In multivariate analyses conducted separately by sex, age ≥55 years was significantly associated with lower values of GI (β = −1.26, *p* < 0.001 in males; β = −1.58, *p* < 0.001 in females) and GL (β = −1.77, *p* = 0.020 in males; β = −2.58, *p* < 0.001 in females). Higher educational level was also inversely related to GI and GL. In males, intermediate (β = −1.04, *p* = 0.005) and high education (β = −2.23, *p* < 0.001) were associated with lower GI, while corresponding estimates for GL were β = −1.43 (*p* = 0.047) and β = −4.80 (*p* < 0.001). Among females, intermediate and high education were similarly associated with lower GI (β = −1.11, *p* = 0.008; β = −2.20, *p* < 0.001) and GL (β = −2.16, *p* < 0.001; β = −4.19, *p* < 0.001). Smoking was positively associated with both indices (β = 1.32, *p* < 0.001 in males; β = 0.68, *p* = 0.053 in females, for GI; and β = 3.93, *p* < 0.001; β = 1.73, *p* < 0.001, respectively, for GL).

Associations with adherence to the Mediterranean diet differed by sex. In males, medium and high adherence were linked to lower GI (β = −1.22, *p* = 0.002; β = −1.20, *p* = 0.033) and lower GL (β = −2.66, *p* < 0.001; β = −2.72, *p* = 0.007), whereas in females, no significant associations were observed for GI and higher adherence was related to higher GL (Table 5 and Table 6).

## 4. Discussion

In a representative sample of the Greek population, we estimated that the mean GI was 59.7 and the mean GL 101.7. GI was stable between subgroups, while the GL was higher in males, especially in underweight and those with intermediate education. Females who followed the Mediterranean diet had a higher GL. Compared to earlier results from EPIC, we found slightly higher GI values in both males (58.3 in EPIC vs. 61.2 in HYDRIA) and females (56.2 in EPIC vs. 59.2 in HYDRIA), and slightly lower GL in both males (119.8 in EPIC vs. 112.9 in HYDRIA) and females (88.7 in EPIC vs. 81.9 in HYDRIA) [32]. Considering that low GI is below 55 and high GI above 70, the average daily GI of the Greek population is intermediate.

After adjusting for carbohydrates, fat and total energy intake, older age and higher educational status were associated with lower GI and GL, while current smoking with higher GI and GL in both men and women. Older age had been associated with higher GI and GL in other studies [33]. Nevertheless, our results are in accordance with previous research reporting that older individuals and those with higher education have lower GI/GL, even after controlling for energy and macronutrients [34]. It is plausible to find a lower dietary GI among older adults as they may still follow a traditional Mediterranean diet which includes higher fiber foods and minimally processed food which tend to have lower GI values [35]. This would also explain our finding of higher GI associated with higher energy and lower fiber intake. The observed association with smoking could be due to the general attitude of smokers towards unhealthy dietary choices. Smoking has been independently associated with unfavourable glycemic control in previous research [36] therefore for this population it would be even more relevant to consume low GI foods which, by definition, result in lower metabolic burden. This highlights also the importance of integrating smoking cessation efforts into strategies aimed at improving metabolic health.

Globally, dietary GI and GL vary considerably, primarily due to differences in culinary habits and dietary patterns shaped by cultural traditions and food processing practices. Cross-national comparisons further highlight the variability and GL values. Our estimates are close to those of other European countries, but even closer to the values reported in the Middle East. Indeed, The EPIC study found lower average GI and GL (127 g/day) across eight European countries, reflecting differences on the consumption of foods such as bread, pasta, legumes and sweets [32]. According to a cross-sectional survey in Greater Beirut, the mean dietary GI in Lebanese adults was 59.9 ± 8 and GL around 209.7 ± 100 [37]. The average dietary GI and GL in the U.S. was 56 and 133, respectively [38]. The last assessment of the national nutrition surveys in Australia reported a dietary GI of 53.9 and GL of 123–135 among adults [39], explained primarily by the consumption of mixed grains and dietary fiber [40].

In contrast, Asian populations exhibit distinct GI/GL profiles, influenced heavily by staple food consumption and traditional cooking methods. For instance, the Chinese diet, which is rich in white rice, typically results in higher dietary GI and GL. A previous study showed that the contribution of carbohydrate to total energy in Chinese people was higher, by 3.1% in 18–64 years old and 5% in ≥65 years old, than that of Italian participants [41]. A further analysis from the China Health and Nutrition Survey reports the average GI and GL values of above 70 and 200, respectively, where participants with higher dietary GI or GL were more likely to have higher intakes of carbohydrate and to consume less total dietary fiber, fat, and protein than those with lower GI or GL [42,43]. In Japan, GI and GL were slightly lower than in China. The National Health and Nutrition Survey reported a mean dietary GI of approximately 65–66 and a GL ranging from 149 to 190 g/day, with white rice contributing over 50% to both measures [44]. In Greece, the main contributor to the GL is bread (40% among men and 31% among women). Fruits (12% and 14%), pasta, rice and grains (9% and 8%), sugar and confectionary (7% and 9%), pastries (5% and 9%) and vegetables (5% and 5%), also contribute to the GL [32]. These regional variations underscore the significance of culturally specific dietary habits in shaping glycemic responses and their subsequent health implications.

Our results also indicate that higher adherence to the Mediterranean diet was associated with lower GI and GL in males, albeit not in women. In the MEDGI-Carb trial, females showed significantly higher 8-h plasma glucose on a high-GI (i.e. >70) Mediterranean Diet, pointing to gender-specific dietary choices, within the Mediterranean diet frame [45]. On the other hand, a low-GI (i.e. <55) Mediterranean pattern improves glycemic variability overall [46].

The Greek Mediterranean diet is particularly rich in olive oil and it generally includes smaller quantities of carbohydrates, a fact that can potentially reduce its GL [31]. Previous research indicates similar average GI in Greece, Italy and Spain, ranging from 52.4 in females from Asturias, Spain to 58.3 in males in Greece. However, GL was lower in Greece, in both males and females, compared to Spain (132.1–149.1 in males and 96.1–110.6 in women) and to Italy (171.4–184.5 in males and 119.3–124.9 in women) [32].

Numerous studies from Western countries, including the United States, Canada and Australia indicate that higher dietary GI and GL are associated with increased risks of metabolic risk, type 2 diabetes and cardiovascular diseases [47,48,49].

Recent meta-analyses of prospective cohort studies have demonstrated that higher dietary GI and GL are consistently associated with increased risk of type 2 diabetes [50] while interventions with low GI diets in people with type 2 improved glycemic control and cardiovascular risk factors [51,52]. Interestingly, a dose-response meta-analysis reported that high GI diets were associated with greater all-cause mortality among women, suggesting potential sex-specific susceptibility to the adverse metabolic effects of high GI dietary patterns [53]. In women with polycystic ovary syndrome, lower GI and GL diets have been shown to improve insulin sensitivity, lipid metabolism, and androgen profiles, representing a valuable non-pharmacological strategy for mitigating both reproductive and metabolic complications [54]. Likewise, in gestational diabetes mellitus, lower GI and GL dietary patterns are associated with improved glycemic control and maternal-fetal outcomes, reinforcing the need to consider glycemic impact during pregnancy [55]. From a public health perspective, these data suggest that creating a national dietary GI and GL database, could be particularly relevant for Greece, where obesity is increasingly prevalent and adherence to the traditional Mediterranean diet continues to decline. Emphasising traditional Greek foods naturally characterised by lower glycemic responses, such as legumes, whole grains, fruits, and nuts, while discouraging refined, high-GI staples may enhance metabolic health outcomes at the population level [23].

Thus, along with previous research, our findings emphasize the necessity of population-specific assessments of these dietary indices, focusing on obese, sedentary people, who tend to have a higher risk of insulin resistance, as well as people with diabetes, PCOS and smokers. Differences in staple carbohydrate types, dietary fiber content, and overall meal composition can substantially influence GI and GL exposures. Reinforcing the traditional Mediterranean dietary model, however with explicit attention to carbohydrate quality, may therefore provide a culturally grounded strategy to reduce the burden of type 2 diabetes and related metabolic disorders in the Greek population.

As such, further national-level investigations are essential to understand the role of dietary GI and GL profiles in shaping public health outcomes. The present focus on Greece contributes to this growing body of evidence by offering detailed insights into GI and GL within a Mediterranean dietary context, thereby enriching global understanding of diet-disease relationships across diverse nutritional settings.

## 5. Strengths and Limitations

A major strength of HYDRIA is the large, nationally representative sample of Greek adults, which permits the generalizability of our findings. Our study includes detailed dietary data, allowing for accurate estimation of GI and GL, as well as comprehensive demographic/individual information that supports subgroup analyses. However, HYDRIA is a survey that mainly includes self-reported data, which are often subject to recall or social desirability bias. Nonetheless, the fact that data collection was performed by specialized personnel mitigates the risk of biases. Another caveat is that only the two 24 h recalls were used to derive the average daily dietary GI and GL. Furthermore, the GI values were derived from international tables and not from local GI testing of staple carbohydrate foods consumed in Greece. Overall, the robust design and population representativeness makes our study one of the most comprehensive investigations of its kind in the Greek population.

## Figures and Tables

**Table 1:** Descriptive Characteristics of Participants, Overall and Stratified by Sex.

	Total *n* = 3951 *n* (%)	Females *n* = 2115 (52%) *n* (%)	Males *n* = 1836 (48%) *n* (%)
*Age group*			
18–34 years	968 (24.3)	519 (23.2)	449 (25.5)
35–44 years	757 (18.3)	408 (17.7)	349 (18.9)
45–54 years	742 (17.5)	402 (17.1)	340 (17.9)
55–64 years	669 (14.9)	361 (15.1)	308 (14.7)
65+ years	806 (25)	416 (26.9)	390 (23)
*BMI category*			
Underweight (<20 kg/m^2^)	121 (2.7)	105 (4.4)	16 (0.9)
Normal (20–24.9 kg/m^2^)	1007 (25)	646 (28.1)	361 (21.6)
Overweight (25–29.9 kg/m^2^)	1491 (37.4)	666 (31.9)	825 (43.5)
Obese (≥30 kg/m^2^)	1266 (34.8)	670 (35.6)	596 (34)
*Educational level*			
Low	1116 (39.6)	644 (44)	472 (34.9)
Intermediate	1613 (38.3)	877 (35.7)	736 (41.2)
High	1222 (22)	594 (20.3)	628 (23.9)
*Employment status*			
Employed	1641 (41.5)	707 (31.7)	934 (52)
All other	2310 (58.5)	1408 (68.3)	902 (48)
*Smoking*			
Never	1647 (43)	1080 (54.9)	567 (30.2)
Ex smoker	840 (21.2)	290 (12.8)	550 (30.3)
Current smoker	1452 (35.8)	741 (32.3)	711 (39.5)
*Med. Diet Adherence Category*			
Low	1725 (44.9)	997 (48.2)	728 (41.4)
Med	1622 (40.1)	856 (38.9)	766 (41.4)
High	604 (15)	262 (12.8)	342 (17.3)

**Table 5:** Results of Multivariate Linear Regression Analysis for Glycemic Index.

	Males		Females	
	β * (95% CI)	*p*-Value	β * (95% CI)	*p*-Value
*Age group*				
18–54 years	0		0	
55+ years	−1.26 (−2.06 to −0.46)	0.002	−1.58 (−2.25 to −0.90)	<0.001
*Educational level*				
Low	0		0	
Intermediate	−1.04 (−1.75 to −0.33)	0.005	−1.11 (−1.92 to −0.30)	0.008
High	−2.23 (−2.95 to −1.50)	<0.001	−2.20 (−3.09 to −1.32)	<0.001
*Smoking*				
Never	0		0	
Ex smoker	−0.25 (−1.12 to 0.61)	0.564	−0.59 (−1.46 to 0.29)	0.185
Current smoker	1.32 (0.59 to 2.05)	<0.001	0.68 (−0.01 to 1.36)	0.053
*Med. Diet Adherence Category*				
Low	0			
Med	−1.22 (−1.98 to −0.47)	0.002		
High	−1.20 (−2.30 to −0.10)	0.033		

* Regression coefficients β correspond to changes on Glycemic index levels associated with one level change on the corresponding covariate.

**Table 6:** Results of Multivariate Linear Regression Analysis for Glycemic Load.

	Males		Females	
	β * (95% CI)	*p*-Value	β * (95% CI)	*p*-Value
*Age group*				
18–54 years	0		0	
55+ years	−1.77 (−3.27 to −0.28)	0.02	−2.58 (−3.75 to −1.41)	<0.001
*Educational Level*				
Low	0			
Med	−1.43 (−2.84 to −0.02)	0.047	−2.16 (−3.55 to −0.77)	0.003
High	−4.80 (−6.28 to −3.32)	<0.001	−4.19 (−5.77 to −2.61)	<0.001
*Smoking*				
Never	0		0	
Ex smoker	0.00 (−1.70 to 1.70)	0.997	−1.04 (−2.63 to 0.55)	0.197
Current smoker	3.93 (2.34 to 5.51)	<0.001	1.73 (0.79 to 2.67)	<0.001
*Med. Diet Adherence Category*				
Low	0			
Med	−2.66 (−4.07 to −1.24)	<0.001		
High	−2.72 (−4.69 to −0.75)	0.007		

* Regression coefficients β correspond to changes on Glycemic load levels associated with one level change on the corresponding covariate.

## Data Availability

The anonymised data can be accessed after approval by the PI (Prof. A.T.) upon reasonable request.

## References

[1] Augustin L.S.A., Kendall C.W.C., Jenkins D.J.A., Willett W.C., Astrup A., Barclay A.W., Björck I., Brand-Miller J.C., Brighenti F., Buyken A.E. (2015). Glycemic index, glycemic load and glycemic response: An International Scientific Consensus Summit from the International Carbohydrate Quality Consortium (ICQC). Nutr. Metab. Cardiovasc. Dis. NMCD.

[2] Foster-Powell K., Holt S.H.A., Brand-Miller J.C. (2002). International table of glycemic index and glycemic load values: 2002. Am. J. Clin. Nutr..

[3] Jenkins D.J., Wolever T.M., Taylor R.H., Barker H., Fielden H., Baldwin J.M., Bowling A.C., Newman H.C., Jenkins A.L., Goff D.V. (1981). Glycemic index of foods: A physiological basis for carbohydrate exchange. Am. J. Clin. Nutr..

[4] FAO/WHO (2007). Carbohydrates in Human Nutrition.

[5] Wolever T., Jenkins D., Jenkins A., Josse R. (1991). The glycemic index: Methodology and clinical implications. Am. J. Clin. Nutr..

[6] Bornet F.R., Costagliola D., Rizkalla S.W., Blayo A., Fontvieille A.M., Haardt M.J., Letanoux M., Tchobroutsky G., Slama G. (1987). Insulinemic and glycemic indexes of six starch-rich foods taken alone and in a mixed meal by type 2 diabetics. Am. J. Clin. Nutr..

[7] Jenkins D.J., Wolever T.M., Collier G.R., Ocana A., Rao A.V., Buckley G., Lam Y., Mayer A., Thompson L.U. (1987). Metabolic effects of a low-glycemic-index diet. Am. J. Clin. Nutr..

[8] Salmerón J. (1997). Dietary Fiber, Glycemic Load, and Risk of Non—Insulin-dependent Diabetes Mellitus in Women. JAMA J. Am. Med. Assoc..

[9] Trumbo P.R. (2021). Global evaluation of the use of glycaemic impact measurements to food or nutrient intake. Public Health Nutr..

[10] Liu S., Willett W.C., Stampfer M.J., Hu F.B., Franz M., Sampson L., Hennekens C.H., Manson J.E. (2000). A prospective study of dietary glycemic load, carbohydrate intake, and risk of coronary heart disease in US women. Am. J. Clin. Nutr..

[11] Ludwig D.S. (2002). The Glycemic Index: Physiological Mechanisms Relating to Obesity, Diabetes, and Cardiovascular Disease. JAMA.

[12] Pawlak D.B., Kushner J.A., Ludwig D.S. (2004). Effects of dietary glycaemic index on adiposity, glucose homoeostasis, and plasma lipids in animals. Lancet.

[13] Augustin L., Franceschi S., Jenkins D., Kendall C., La Vecchia C. (2002). Glycemic index in chronic disease: A review. Eur. J. Clin. Nutr..

[14] Ebbeling C.B., Leidig M.M., Feldman H.A., Lovesky M.M., Ludwig D.S. (2007). Effects of a low-glycemic load vs low-fat diet in obese young adults: A randomized trial. JAMA.

[15] Meng H., Matthan N.R., Ausman L.M., Lichtenstein A.H. (2017). Effect of macronutrients and fiber on postprandial glycemic responses and meal glycemic index and glycemic load value determinations. Am. J. Clin. Nutr..

[16] Silva F.M., Kramer C.K., Crispim D., Azevedo M.J. (2015). A High–Glycemic Index, Low-Fiber Breakfast Affects the Postprandial Plasma Glucose, Insulin, and Ghrelin Responses of Patients with Type 2 Diabetes in a Randomized Clinical Trial1–3. J. Nutr..

[17] Trichopoulou A., Costacou T., Bamia C., Trichopoulos D. (2003). Adherence to a Mediterranean diet and survival in a Greek population. N. Engl. J. Med..

[18] Inomaki R., Murakami K., Livingstone M.B.E., Okubo H., Kobayashi S., Suga H., Sasaki S. (2017). A Japanese diet with low glycaemic index and glycaemic load is associated with both favourable and unfavourable aspects of dietary intake patterns in three generations of women. Public Health Nutr..

[19] Castro M.A., Carlos J.V., Lopes R.C.V., Januário B.L., Marchioni D.M.L., Fisberg R.M. (2014). Dietary Glycemic Index, Glycemic Load, and Nutritional Correlates in Free-Living Elderly Brazilians: A Population-Based Survey. J. Am. Coll. Nutr..

[20] Hills A.P., Arena R., Khunti K., Yajnik C.S., Jayawardena R., Henry C.J., Street S.J., Soares M.J., Misra A. (2018). Epidemiology and determinants of type 2 diabetes in south Asia. Lancet Diabetes Endocrinol..

[21] Keys A., Keys M. (1975). How to Eat Well and Stay Well the Mediterranean Way.

[22] Rossi M., Turati F., Lagiou P., Trichopoulos D., Augustin L.S., La Vecchia C., Trichopoulou A. (2013). Mediterranean diet and glycaemic load in relation to incidence of type 2 diabetes: Results from the Greek cohort of the population-based European Prospective Investigation into Cancer and Nutrition (EPIC). Diabetologia.

[23] Papakonstantinou E., Galanopoulos K., Kapetanakou A.E., Gkerekou M., Skandamis P.N. (2022). Short-Term Effects of Traditional Greek Meals: Lentils with Lupins, Trahana with Tomato Sauce and Halva with Currants and Dried Figs on Postprandial Glycemic Responses—A Randomized Clinical Trial in Healthy Humans. Int. J. Environ. Res. Public Health.

[24] Sievenpiper J.L. (2012). Fructose: Where Does the Truth Lie?. J. Am. Coll. Nutr..

[25] Martínez-González M.A., Salas-Salvadó J., Estruch R., Corella D., Fitó M., Ros E. (2015). Benefits of the Mediterranean Diet: Insights from the PREDIMED Study. Prog. Cardiovasc. Dis..

[26] Moreiras-Varela O. (1989). The Mediterranean diet in Spain. Eur. J. Clin. Nutr..

[27] Augustin L.S., Ellis P.R., Vanginkel M.-A., Riccardi G. (2025). Pasta: Is It an Unhealthy Refined Food?. J. Nutr..

[28] D’Alessandro A., De Pergola G. (2014). Mediterranean Diet Pyramid: A Proposal for Italian People. Nutrients.

[29] European Food Safety Authority (2014). Guidance on the EU Menu methodology. EFSA J..

[30] European Health Examination Survey (2016). EHES Manuals: General Guidelines and Examination Protocols (Parts A–F).

[31] Martimianaki G., Peppa E., Valanou E., Papatesta E.M., Klinaki E., Trichopoulou A. (2022). Today’s Mediterranean Diet in Greece: Findings from the National Health and Nutrition Survey—HYDRIA (2013–2014). Nutrients.

[32] Van Bakel M.M.E., Kaaks R., Feskens E.J.M., Rohrmann S., Welch A.A., Pala V., Avloniti K., Van Der Schouw Y.T., Van Der A D.L., Du H. (2009). Dietary glycaemic index and glycaemic load in the European Prospective Investigation into Cancer and Nutrition. Eur. J. Clin. Nutr..

[33] Różańska D., Waśkiewicz A., Regulska-Ilow B., Kwaśniewska M., Pająk A., Stepaniak U., Kozakiewicz K., Tykarski A., Zdrojewski T.R., Drygas W. (2019). Relationship between the dietary glycemic loadof the adult Polish population and socio-demographicand lifestyle factors—Results of the WOBASZ II study. Adv. Clin. Exp. Med..

[34] Sahyoun N.R., Anderson A.L., Kanaya A.M., Koh-Banerjee P., Kritchevsky S.B., de Rekeneire N., Tylavsky F.A., Schwartz A.V., Lee J.S., Harris T.B. (2005). Dietary glycemic index and load, measures of glucose metabolism, and body fat distribution in older adults. Am. J. Clin. Nutr..

[35] Rodríguez-Rejón A.I., Castro-Quezada I., Ruano-Rodríguez C., Ruiz-López M.D., Sánchez-Villegas A., Toledo E., Artacho R., Estruch R., Salas-Salvadó J., Covas M.I. (2014). Effect of a Mediterranean Diet Intervention on Dietary Glycemic Load and Dietary Glycemic Index: The PREDIMED Study. J. Nutr. Metab..

[36] Sia H.-K., Kor C.-T., Tu S.-T., Liao P.-Y., Wang J.-Y. (2022). Association between smoking and glycemic control in men with newly diagnosed type 2 diabetes: A retrospective matched cohort study. Ann. Med..

[37] Borgi C., Taktouk M., Nasrallah M., Isma’eel H., Tamim H., Nasreddine L. (2020). Dietary Glycemic Index and Glycemic Load Are Not Associated with the Metabolic Syndrome in Lebanese Healthy Adults: A Cross-Sectional Study. Nutrients.

[38] Della Corte K.A., Della Corte D., Titensor S., Yang B., Liu S. (2024). Development of a national database for dietary glycemic index and load for nutritional epidemiologic studies in the United States. Am. J. Clin. Nutr..

[39] Kusnadi D.T.L., Barclay A.W., Brand-Miller J.C., Louie J.C.Y. (2017). Changes in dietary glycemic index and glycemic load in Australian adults from 1995 to 2012. Am. J. Clin. Nutr..

[40] Buyken A.E., Goletzke J., Joslowski G., Felbick A., Cheng G., Herder C., Brand-Miller J.C. (2014). Association between carbohydrate quality and inflammatory markers: Systematic review of observational and interventional studies. Am. J. Clin. Nutr..

[41] Zhang R., Wang Z., Fei Y., Zhou B., Zheng S., Wang L., Huang L., Jiang S., Liu Z., Jiang J. (2015). The Difference in Nutrient Intakes between Chinese and Mediterranean, Japanese and American Diets. Nutrients.

[42] Li M., Cui Z., Meng S., Li T., Kang T., Ye Q., Cao M., Bi Y., Meng H. (2020). Associations between Dietary Glycemic Index and Glycemic Load Values and Cardiometabolic Risk Factors in Adults: Findings from the China Health and Nutrition Survey. Nutrients.

[43] Yu D., Zhang X., Shu X.-O., Cai H., Li H., Ding D., Hong Z., Xiang Y.-B., Gao Y.-T., Zheng W. (2016). Dietary glycemic index, glycemic load, and refined carbohydrates are associated with risk of stroke: A prospective cohort study in urban Chinese women. Am. J. Clin. Nutr..

[44] Murakami K., Sasaki S. (2018). Glycemic index and glycemic load of the diets of Japanese adults: The 2012 National Health and Nutrition Survey, Japan. Nutrition.

[45] Vitale M., Costabile G., Bergia R.E., Hjorth T., Campbell W.W., Landberg R., Riccardi G., Giacco R. (2023). The effects of Mediterranean diets with low or high glycemic index on plasma glucose and insulin profiles are different in adult men and women: Data from MEDGI-Carb randomized clinical trial. Clin. Nutr..

[46] Bergia R.E., Giacco R., Hjorth T., Biskup I., Zhu W., Costabile G., Vitale M., Campbell W.W., Landberg R., Riccardi G. (2022). Differential Glycemic Effects of Low- versus High-Glycemic Index Mediterranean-Style Eating Patterns in Adults at Risk for Type 2 Diabetes: The MEDGI-Carb Randomized Controlled Trial. Nutrients.

[47] Brand-Miller J.C. (2003). Glycemic Load and Chronic Disease. Nutr. Rev..

[48] Clar C., Al-Khudairy L., Loveman E., Kelly S.A., Hartley L., Flowers N., Germanò R., Frost G., Rees K. (2017). Low glycaemic index diets for the prevention of cardiovascular disease. Cochrane Database Syst. Rev..

[49] Hu F.B., Manson J.E., Stampfer M.J., Colditz G., Liu S., Solomon C.G., Willett W.C. (2001). Diet, Lifestyle, and the Risk of Type 2 Diabetes Mellitus in Women. N. Engl. J. Med..

[50] Livesey G., Taylor R., Livesey H.F., Buyken A.E., Jenkins D.J.A., Augustin L.S.A., Sievenpiper J.L., Barclay A.W., Liu S., Wolever T.M.S. (2019). Dietary Glycemic Index and Load and the Risk of Type 2 Diabetes: A Systematic Review and Updated Meta-Analyses of Prospective Cohort Studies. Nutrients.

[51] Jenkins D.J.A., Kendall C.W.C., McKeown-Eyssen G., Josse R.G., Silverberg J., Booth G.L., Vidgen E., Josse A.R., Nguyen T.H., Corrigan S. (2008). Effect of a Low–Glycemic Index or a High–Cereal Fiber Diet on Type 2 Diabetes: A Randomized Trial. JAMA.

[52] Jenkins D.J.A., Kendall C.W.C., Augustin L.S.A., Mitchell S., Sahye-Pudaruth S., Blanco Mejia S., Chiavaroli L., Mirrahimi A., Ireland C., Bashyam B. (2012). Effect of Legumes as Part of a Low Glycemic Index Diet on Glycemic Control and Cardiovascular Risk Factors in Type 2 Diabetes Mellitus: A Randomized Controlled Trial. Arch. Intern. Med..

[53] Shahdadian F., Saneei P., Milajerdi A., Esmaillzadeh A. (2019). Dietary glycemic index, glycemic load, and risk of mortality from all causes and cardiovascular diseases: A systematic review and dose-response meta-analysis of prospective cohort studies. Am. J. Clin. Nutr..

[54] Manta A., Paschou S., Isari G., Mavroeidi I., Kalantaridou S., Peppa M. (2023). Glycemic Index and Glycemic Load Estimates in the Dietary Approach of Polycystic Ovary Syndrome. Nutrients.

[55] Mavroeidi I., Manta A., Asimakopoulou A., Syrigos A., Paschou S.A., Vlachaki E., Nastos C., Kalantaridou S., Peppa M. (2024). The Role of the Glycemic Index and Glycemic Load in the Dietary Approach of Gestational Diabetes Mellitus. Nutrients.

